# Basic biochemical and hematological parameters of structural hemoglobin variants in the postpartum women and their respective newborn from Manaus, Amazonas, Brazil

**DOI:** 10.1186/s12884-022-05143-7

**Published:** 2022-12-15

**Authors:** Roberta da Silva Brito, Lecita Marreira de Lima Barros, Lilian Wallace Moreira, Regina Neves Normando, Thiago Bacha de Jesus, Marilda de Souza Gonçalves, Rajendranath Ramasawmy, Stéfani Ferreira de Oliveira, Keyla Emanulle Ramos da Silva, Nelson Abrahim Fraiji, Larissa Feitosa da Hora, Rebeca Linhares de Abreu Netto, José Pereira de Moura Neto

**Affiliations:** 1 Fundação Hospitalar de Hematologia e Hemoterapia do Amazonas, Manaus, Amazonas Brazil; 2 Universidade Federal do Amazonas, Faculdade de Ciências Farmacêuticas, General Rodrigo Otávio Jordão Ramos Avenue, 6200 - Coroado I, Manaus, Amazonas CEP: 69067-005 Brazil; 3 Fundação Oswaldo Cruz - Centro de Pesquisas Gonçalo Moniz, Salvador, Bahia Brazil; 4 Fundação de Medicina Tropical Dr. Heitor Vieira Dourado, Manaus, Amazonas Brazil

**Keywords:** Structural hemoglobinopathies, Postpartum women, Newborns, Manaus

## Abstract

The present study aimed at estimating the prevalence of structural hemoglobinopathies in newborn and describing the hematological and biochemical characteristics between postpartum women (PW) and their respective newborns (NB) at a public maternity hospital in Manaus, Amazonas state, Brazil. In total, 825 NB and 820 PW were included in the study. Hematological and biochemical analysis and screening of structural hemoglobinopathies were performed and compared in groups of individuals (NICU or not; hemoglobin genotypes; gestational age and prenatal). The age of PW ranged from 13 to 44 years old (mean of 23.7 ± 6.6 years), with 45.9% pregnant for the first time and 54.1% multiparous. Reported receiving prenatal care 88% and regarding the type of delivery, 47.7% had delivered by cesarean section. Among the births, 19.4% were born premature and 8.3% were admission to the neonatal intensive care unit (NICU). The male NB represented 53.4% of the total. Sickle cell trait (FAS) was found in 16 (1.94%) and heterozygous for D hemoglobin (FAD) in 6 (0.73%) newborns. A statistically significant values was found between the previous history of miscarriage and increase of Mean corpuscular volume (MCV) (*p* < .001), Red blood cell distribution width (RDW) (*p* = .003), total and indirect bilirubin concentration (*p* < .001) and LDL cholesterol (*p* = .004). Hemoglobin levels below 13.5 g/dL was found in 66% black newborns, compared with 15% of Afro-Brazilian and 5% of whites. The frequency of structural hemoglobinopathies was higher in African-Brazilian newborn babies (78%) and those who with low birth weight had a higher frequency of NICU (35.7%). Interestingly, underage mothers had a higher frequency of NB with low birth weight and premature birth. Postpartum women who had children carriers of FAS and FAD had a higher frequency of urinary tract infection (65.2%) and moderate anemia (23.8%). This study estimated for the first time the prevalence of structural hemoglobinopathies in NB in Manaus, Amazonas, Brazil. Despite the small prevalence of, we highlight the importance of early diagnosis of hemoglobin variants, contributing to the improvement of the quality of life of PW and your NB, reinforce the need to implement educational and prevention programs to raise awareness among the population and in order to counsel parents regarding the probability of having a child with abnormal hemoglobins homozygous as HbSS or HbCC.

**Supplementary Information:**

The online version contains supplementary material available at 10.1186/s12884-022-05143-7.

## Introduction

Structural hemoglobinopathies are caused by simple substitutions, small insertions or deletions of nucleotides, which may subsequently change the amino acids in a protein. These alterations may compromise physicochemical properties such as electric charges, solubility, molecular stability and oxygen affinity, depending on the type of mutation and its location [1].

By September, 2014, more than 1200 mutations in the genes of alpha and beta chains of the hemoglobin molecule had already been described in the Globin Gene Server database (http://globin.cse.psu.edu/). Most of the described abnormal hemoglobin do not cause clinical symptoms; however, they may be associated with relevant pathophysiology [2].

According to the World Health Organization (WHO), hemoglobin disorders affect approximately 5.5% of the world population. These are common in 71% of the 229 countries and it is estimated that 270 million people are the carrier of genes that determine the presence of abnormal hemoglobin. In addition, it is estimated that 300 to 400 thousand children are born each year with sickle cell anemia or severe thalassemia. Of these, 3.4% die before reaching the age of five and this percentage can reach up to 6.4% in Africa [3, 4].

The introduction of variant hemoglobins, notably hemoglobinopathy S, took place with greater intensity during the Brazilian slave period of slaves originating from the Sudanese and Bantu groups, who entered Brazil through the ports of Rio de Janeiro, Pernambuco and Bahia. Studies carried out in different regions of Brazil have shown that among the abnormal hemoglobin, the structural abnormal hemoglobins S (HbS) and HbC of African origin have the highest frequency. This demonstrates the intense participation of people of African descent in the composition of Brazilian population [5–7].

Despite its higher prevalence in African-Brazilian, population studies have shown an increased presence of HbS in white individuals. In the Afro-Brazilians, the average prevalence of AS heterozygotes is 2%; a number that reaches up to 6 to 10%, while the prevalence of AC heterozygotes is 1 to 3% [8]. In Brazil, approximately 2000 children are born with sickle cell anemia each year, 50,000 patients are born with hemoglobin S and more than 20,000 with other abnormal hemoglobin (C, D or E). Studies conducted in Brazil have revealed that there are approximately 10 million individuals with abnormal hemoglobin, and that annually about 3000 are born with the homozygous form [9].

Thus, the present study aimed at estimating the prevalence of structural hemoglobinopathies in newborn, and describing the hematological and biochemical characteristics between postpartum women and their respective newborns at a public maternity hospital in Manaus, Amazonas state, Brazil.

## Participants and methods

### Participants

The study population sample was composed of 820 postpartum women and their 825 respective newborns (five mothers had twins), from Manaus, capital of the state of Amazonas, Brazil, who were attended at the Instituto da Mulher Dona Lindu (IMDL) during the period between March, 2014 and January, 2015. All postpartum women answered a questionnaire concerning personal data and their current and previous obstetric history. In the case of the underage postpartum women, the consent form was signed by the parent or guardian.

All participants, regardless of the type of childbirth (normal or cesarean) and gestational age (term or preterm – in weeks), agreed to participate in the study and signed the consent form. Postpartum women with mental disorders were excluded from the study.

Information regarding the ethnicity of newborn was collected from the mother (who was asked what her colour and of the child’s father). Both parents of black colour, we classified your newborn as blacks; mother and father whites, your newborn as white; mother/father black, white and or brown, your newborn as African-Brazilian.

### Clinical parameters

Complementary data regarding the newborns were obtained through their medical records and questionnaires applied to postpartum women. These questionnaires probed variables such ethnicity, type of nutrition, birth weight, gender; skin color; twinning; received blood transfusions and hospital admission (neonatal intensive care unit-NICU). To postpartum women the questionnaire had gestational age, prenatal care, type of delivery, fetal death, previous history of miscarriage, smoking, family anemia and diabetes.

### Hematological and biochemical analysis

Peripheral blood samples from the pregnant women were collected the day before delivery, while from the newborn on the same day of their birth during a routine follow-up visit. Hematological analyses were performed using an automated hematologic analyzer ADVIA 120 (Siemens Healthineers Brasil). Data obtained included total red blood cell (RBC) count, hemoglobin concentration, hematocrit, mean corpuscular volume (MCV), mean corpuscular hemoglobin (MCH), mean corpuscular hemoglobin concentration (MCHC), red blood cell distribution width (RDW), total and differential leukocyte count and platelet counts. Biochemical analyses were performed using an automated biochemical analyzer A25 (BioSystems SA, Barcelona, Spain). The parameters analyzed included: liver profile (total bilirubin and fractions); iron studies (serum iron, ferritin, transferrin and iron binding capacity); lipid profile (total cholesterol, cholesterol fractions and triglycerides); renal profile (urea and creatinine); lactate dehydrogenase (LDH) and glucose.

Analysis of the newborn data, established < 2500 g as being underweight and > 2500 g the normal weight and hemoglobin < 13.5 g/dL as anemic and ≥ 13.5 g/dL normal according to WHO. Postpartum women with hemoglobin levels lower than 11 g/dL were classified as anemic.

### Hemoglobin analysis

Hemoglobin profiles were characterized using high-performance liquid chromatography (HPLC) (Bio-Rad, Hercules, CA, USA). HPLC analyses were interpreted using AFCS control hemoglobins, utilizing the sickle cell kit for hemoglobin screening. The laboratory diagnosis of sickle cell disease is based on the detection of hemoglobin S and should follow the standards established in the National Neonatal Screening Program (Ministry of Health Ordinance No. 822/01) [10, 11].

### Data analysis

Statistical analysis was performed using IBM SPSS 23 (Armonk, NY) and GraphPad Prism 5.0 (San Diego, CA) softwares. To evaluate the distributions of the studied variables was performed using the Kolmogorov-Smirnov test. The ANOVA parametric test was used to compare quantitative variables with normal distribution and non-parametric Kruskal-Wallis test the off-normal distributions. The analysis of qualitative or categorical variables of three or more groups was performed by nonparametric chi-square (χ2), corrected by the Mantel-Haenszel test and Yates. *P*-values < 0.05 were considered significant.

## Results

Data on frequency of births by gestational age and of pregnancies among postpartum women interviewed at IMDL are described in Table 1. The age of postpartum women ranged from 13 to 44 years old, with a mean of 23.7 ± 6.6. Regarding the number of pregnancies, it was observed that 375 (45.73%) were pregnant for the first time, while 445 (54.27%) were multiparous. On average, each woman had three pregnancies. Those with children with Sickle cell trait (FAS) profile, two pregnancies, whose child had Heterozygous for D hemoglobin (FAD), only one pregnancy, and those with child with a Normal Hemoglobin (FAA) profile between 1 and 10 pregnancies.

**Table 1:** Frequency of births by gestational age and pregnancies from postpartum women attended at IMDL/Manaus-AM (March, 2014 – January, 2015)

Gestational Age (Weeks)	Number of births	
	N°	%
28–31	21	2.57
32–35	139	16.95
36 or more	660	80.48
Total	820	100.0
Frequency	Pregnancies	
	N°	%
1	375	45.73
2	224	27.32
3	103	12.56
4	52	6.34
5	31	3.78
6	16	1.95
7	7	0.85
8	5	0.07
9	5	0.07
10	2	0.24
Total	820	100

N°: Total Number

Table 2 summarize and compare the hematological data between gestational ages by weeks. Interestingly, the hematimetric indices were lower in pregnant women with lower gestational age, however, with a significant difference only for RBC (*p* = 0.048). We also found a higher absolute count of monocytes (*p* < 0.001) and eosinophils (*p* = .005) in those pregnant women with a lower gestational age.

**Table 2:** Analysis of hematological data by gestational age in postpartum women attended at IMDL/Manaus-AM (March, 2014 – January, 2015)

Hematological data	Gestational age (weeks)			*p*-value
	28–31 *n* = 30	32–35 *n* = 204	36 or more *n* = 586	
	Means ± SD			
RBC (10^6^/μL)	4.01 ± 0.69	4.28 ± 0.44	4.3 ± 0.41	**.048**
Hemoglobin (g/dl)	12.03 ± 2.12	12.44 ± 1.47	12.5 ± 1.31	.439
Hematocrit (%)	37.30 ± 6.86	38.59 ± 5.52	38.63 ± 4.09	.498
MCV (fL)	90.01 ± 6.13	90.25 ± 6.02	90.07 ± 6.63	.253
MHC (pg)	30.99 ± 1.98	29.14 ± 2.41	29.12 ± 2.26	.651
MCHC (g/dl)	32.30 ± 1.18	32.28 ± 1.44	32.36 ± 1.39	.910
RDW (%)	13.58 ± 1.11	14.01 ± 1.13	14.93 ± 1.26	.575
Leukocytes (/mm^3^)	12,344.7 ± 3318.9	11,938.3 ± 3404.7	12,280.9 ± 3619.4	.727
Neutrophils (/mm^3^)	8943.1 ± 2604.4	9055.7 ± 3167.1	9394.2 ± 3634.7	.669
Lymphocytes (/mm^3^)	1854.9 ± 732.7	1952.6 ± 758.9	1845.7 ± 747.2	.498
Monocytes (/mm^3^)	755.5 ± 151.5	501.6 ± 264.9	426.2 ± 219.6	**<.001**
Eosinophils (/mm^3^)	205.5 ± 75.8	157.2 ± 69.9	165.8 ± 79.5	**.005**
Basophils (/mm^3^)	34.6 ± 35.1	67.9 ± 54.8	52.7 ± 49.2	.097
Platelet (×10^9^/L)	257.3 ± 80.9	243.9 ± 71.6	237.1 ± 65.2	.380
MPV (fL)	7.93 ± 1.32	8.95 ± 1.69	8.78 ± 1.41	.110

Statistically significant associations (*p* < .05) are emphasized in bold type

*RBC* Red blood cell, *MCV* Mean corpuscular volume, *MCH* Mean corpuscular hemoglobin, *MCHC* Mean corpuscular hemoglobin concentration, *RDW* Red blood cell distribution width, *MPV* Mean platelet volume, *SD* Standard Deviation *n* Number of postpartum women

Of the 820 postpartum women studied, 722 (88.05%) reported receiving prenatal care and only 27 (3.3%) had continued smoking during pregnancy. Regarding clinical conditions in postpartum women, 28 (3.4%) had gestational diabetes and the anemia in the family was found in 81 (9.8%) of postpartum women; however, all of them stated that they were unaware of the cause. Our results presented 40 (4.88%) postpartum women developed hypertensive disorders of pregnancy (HDP) and 130 (15.9%) had a previous history of miscarriage. Higher risk of giving birth to a child weighing < 2500 kg was 3.61 times (CI: 2.08–9.65; *p* < 0.001) in puerperal women who developed PDH and 6.01 times (CI: 3.65–10.14: *p* < 0.001) in those with a previous history of miscarriage (Supplementary Table 1). Besides that, postpartum women with a previous history of miscarriage presented increase significant of the concentration of MCV (*p* < .001), RDW (*p* = .003), total and indirect bilirubin (*p* < .001) and all serum lipids, however, with significant values only for LDL cholesterol (*p* = .004) (data no showed).

Regarding the distribution of hemoglobin profile in newborns, the total prevalence found for abnormal hemoglobin was 2.67%, in which 16 (1.94%) was for FAS and 6 (0.73%) for FAD. Tables 3 and 4 show no statistically significant results for hematological and biochemical data among newborn hemoglobin genotypes.

**Table 3:** Distribution of hematological data by hemoglobin profile in newborns from IMDL (March, 2014 – January, 2015)

Hematological data	FAA (*n* = 803)	FAS (*n* = 16)	FAD (*n* = 6)	*p*-value
	Means ± SD			
RBC (10^6^/μL)	4.54 ± 0.63	4.58 ± 0.32	4.32 ± 0.49	.246
Hemoglobin (g/dl)	15.62 ± 2.17	15.65 ± 1.72	14.95 ± 1.89	.534
Hematocrit (%)	51.18 ± 7.14	51.07 ± 5.03	48.52 ± 5.95	.559
MCV (fL)	112.96 ± 7.96	112.19 ± 6.68	123.5 ± 8.32	.551
MHC (pg)	34.45 ± 2.57	34.33 ± 1.96	36.50 ± 2.42	.492
MCHC (g/dl)	30.61 ± 2.32	30.62 ± 1.38	30.34 ± 1.97	.993
RDW (%)	16.20 ± 1.53	16.30 ± 1.26	15.78 ± 1.62	.531
Leukocytes (/mm^3^)	12,535.4 ± 4564.1	12,327.8 ± 4038.4	8940.1 ± 3122.8	.628
Neutrophils (/mm^3^)	5532.9 ± 3489.8	6632.7 ± 2802,6	4908.0 ± 1433.8	.664
Band Neutrophils (/mm^3^)	177.8 ± 102.7	166.5 ± 98.1	98.6 ± 81.5	.169
Lymphocytes (/mm^3^)	5627.5 ± 3292.6	4559.6 ± 2465.1	4074.4 ± 1655.5	.261
Monocytes (/mm^3^)	852.62 ± 605.71	809.8 3 ± 398.84	706.26 ± 15.94	.952
Eosinophils (/mm^3^)	363.78 ± 328.45	271.02 ± 340.18	178.80 ± 5.38	.758
Basophils (/mm^3^)	124.59 ± 96.52	60.96 ± 51.54	71.52 ± 69.6	.436
Platelet (×10^9^/L)	232.68 ± 81.32	261.25 ± 42.53	191.05 ± 1.43	.132
MPV (fL)	9.68 ± 1.36	9.62 ± 1.12	10.09 ± 1.65	.966
Reticulocytes (%)	2.79 ± 0.96	2.74 ± 1.48	3.28 ± 1.59	.478

*FAA* Normal Hemoglobin, *FAS* Sickle cell trait, *FAD* Heterozygous for D hemoglobin, *RBC* Red blood cell, *MCV* Mean corpuscular volume, *MCH* Mean corpuscular hemoglobin, *MCHC* Mean corpuscular hemoglobin concentration, *RDW* Red blood cell distribution width, *SD* Standard Deviation, *n* Number of newborn

**Table 4:** Distribution biochemical data by hemoglobin profile in newborns from IMDL (March, 2014 – January, 2015)

Biochemical data	FAA (*n* = 803)	FAS (*n* = 16)	FAD (*n* = 6)	*p*-value
	Means ± SD			
Urea (mg/dL)	19.56 ± 5.78	15.56 ± 4.87	17.71 ± 4.72	.055
Creatinine (mg/dL)	0.86 ± 5.02	0.586 ± 0.19	0.706 ± 0.29	.943
GGT (mg/dL)	75.13 ± 62.2	45.87 ± 29.59	117.26 ± 71.02	.066
DB (mg/dL)	2.48 ± 2.596	1.88 ± 0.18	2.64 ± 1.17	.943
IB (mg/dL)	2.48 ± 2.59	1.875 ± 0.18	2.64 ± 1.1	.943
TB (mg/dL)	2.017 ± 2.514	1.405 ± 0.134	2.24 ± 1.258	.935
Glucose (mg/dl)	82.02 ± 22.02	70.00 ± 1.38	70.00 ± 1.43	.555
Triglycerides (mg/dL)	38.11 ± 35.7	53.50 ± 58.67	32.66 ± 21.63	.321
HDL (mg/dL)	38.14 ± 12.6	43.49 ± 8.83	36.68 ± 12.86	.332
LDH (μ/l)	1121.5 ± 659.5	1018.5 ± 459.1	899.1 ± 280.7	.617
Iron (mcg/dL)	99.11 ± 63.4	78.44 ± 62.89	105.9 ± 2.78	.627
Ferritin (ng/dL)	94.92 ± 52.1	99.34 ± 27.30	136.2 ± 0.75	.717
Transferrin (mg/dl)	134.77 ± 98.6	69.82 ± 22.21	93.91 ± 1.43	.397
IBC (μg/dL)	421.11 ± 101.8	423.56 ± 5.69	439.09 ± 3.65	.866

*FAA* Normal Hemoglobin, *FAS* Sickle cell trait, *FAD* Heterozygous for D hemoglobin, *GGT* Gamma glutamyl transpeptidase, *DB* Direct bilirubin, *BI* Indirect bilirubin, *TB* Total bilirubin, *HDL* High-density lipoprotein, *LDH* Lactate dehydrogenase, *IBC* Iron binding capacity, *SD* Standard Deviation, *n* Number of newborns

The newborns presented a distribution of 53.4% (440) for males and 46.6% (385) for females. Regarding the type of delivery, 52.3% (429) had normal childbirth, whereas 47.7% (391) were delivered by cesarean section. Among the births, 19.4% (160) were born premature, while 80.6% (660) were full term.

The mean weight according to the hemoglobin profile was 3257.15 g for FAA, 3168.76 g for FAS for and 3433.37 g for FAD. As for racial classification, we noted the predominance of Afro-descendants with a distribution of 81.2% (670) followed by whites with 16.36% (135) and blacks with 2.42% (20). With regard to the admission to the NICU, 91.64% (756) of the newborns did not present any neonatal complications.

In our study, we showed that clinical events, such as hospitalizations in the NICU, were associated with reduced numbers of red blood cells, hemoglobin levels and hematocrit (*p* < 0.001), respectively (Table 5). Interestingly, pregnant women who had a previous history of abortion had the lowest frequency of prenatal care (PR: 1.81–95% CI: 1.25–2.52 - *p* = .002) (Data no showed). It is noteworthy that no significant differences were found in the hematological and biochemical values between the mothers who received prenatal care and those who did not have access to prenatal care (Table 6).

**Table 5:** Hematological data analysis of Neonatal Intensive Care Unit at IMDL (March, 2014 – January, 2015)

Hematological Data	NICU	Means ± SD		*p*-value
RBC × 10^6^/mm^3^	Yes	3.89	0.88	**<.001**
	No	4.68	0.47	
Hemoglobin (g/dl)	Yes	13.28	3.44	**<.001**
	No	16.10	1.74	
Hematocrit (%)	Yes	42.91	12.21	**<.001**
	No	52.70	5.72	
MCV (fL)	Yes	109.0	11.74	.065
	No	112.8	9.51	
MCH (pg)	Yes	33.82	2.47	.214
	No	34.46	2.39	
MCHC (g/dl)	Yes	31.22	2.75	.224
	No	30.64	2.21	
RDW (%)	Yes	16.11	1.33	.546
	No	16.18	1.46	
Leukocytes (/mm^3^)	Yes	12,063.6	5077.0	.358
	No	12,962.6	4418.4	
Neutrophils (/mm^3^)	Yes	4899.2	3343.1	.223
	No	5834.3	3499.0	
Band Neutrophils (/mm^3^)	Yes	170.18	123.75	.559
	No	187.35	133.91	
Lymphocytes (/mm^3^)	Yes	5973.16	4783.25	.720
	No	5713.26	3195.55	
Monocytes (/mm^3^)	Yes	857.84	605.45	.888
	No	874.77	541.86	
Eosinophils (/mm^3^)	Yes	213.52	115.83	.077
	No	377.48	431.77	
Basophils (/mm^3^)	Yes	121.24	105.95	.840
	No	128.16	102.57	
Platelets (×10^9^/L)	Yes	252.82	90.24	.260
	No	233.41	77.58	
MPV (fL)	Yes	9.29	1.56	.162
	No	9.71	1.34	
Reticulocytes (%)	Yes	3.08	0.827	.105
	No	2.71	0.902	

Statistically significant associations (*p* < .05) are emphasized in bold type

*RBC* Red blood cell, *MCV* Mean corpuscular volume, *MCH* Mean corpuscular hemoglobin, *MCHC* Mean corpuscular hemoglobin concentration, *RDW* Red blood cell distribution width, *MPV* Mean platelet volume, *SD* Standard Deviation, *NICU* Neonatal Intensive Care Unit

**Table 6:** Analysis of the hematological data by prenatal care from postpartum women attended at IMDL/Manaus-AM (March, 2014 – January, 2015)

Hematological data	Prenatal	Means ± SD		*p*-value
RBC × 10^6^/mm^3^	Yes	4.26	0.40	.472
	No	4.19	0.41	
Hemoglobin (g/dl)	Yes	12.47	1.23	.614
	No	12.34	1.18	
Hematocrit (%)	Yes	38.36	3.79	.265
	No	37.46	3.50	
MCV (fL)	Yes	90.26	6.21	.499
	No	89.38	4.48	
MCH (pg)	Yes	29.33	1.99	.794
	No	29.44	1.56	
MCHC (g/dl)	Yes	32.51	1.09	.052
	No	33.02	1.03	
RDW (%)	Yes	13.99	1.27	.346
	No	13.74	1.03	
Leukocytes (/mm^3^)	Yes	12,171.5	3431.7	.611
	No	11,806.9	3267.2	
Neutrophils (/mm^3^)	Yes	9267.5	3343.7	.652
	No	8949.9	3432.2	
Lymphocytes (/mm^3^)	Yes	1918.1	805.95	.394
	No	1775.5	660.32	
Monocytes (/mm^3^)	Yes	501.4	296.11	.439
	No	454.2	226.93	
Eosinophils (/mm^3^)	Yes	158.2	177.32	.579
	No	179.0	185.35	
Basophils (/mm^3^)	Yes	61.01	97.50	.837
	No	56.88	68.78	
Platelets (×10^9^/L)	Yes	236.61	64.74	.774
	No	240.48	56.28	
MPV (fL)	Yes	9.03	1.57	.470
	No	8.80	1.05	

*RBC* Red blood cell, *MCV* Mean corpuscular volume, *MCH* Mean corpuscular hemoglobin, *MCHC* Mean corpuscular hemoglobin concentration, *RDW* Red blood cell distribution width, *MPV* Mean platelet volume, *SD* Standard Deviation

Hemoglobin levels below 13.5 g/dL was found in 66% black newborns, compared with 15% of Afro-Brazilian and 5% of whites (Table 7).

**Table 7:** Association between racial classification and hemoglobin values from newborns attended at IMDL/Manaus-AM (March, 2014 – January, 2015)

RACE	Hb ≤ 13.5 g/dL	Hb > 13.5 g/dL	AF vs WH (95% CI)	BLvs WH (95% CI)	BL vs AF (95% CI)
Afro-Brazilian	100 (14.9%)	570 (85,1%)	PR: 2.87 (1.34–6.68) *p* < .001	PR: 12.53 (5.44–27.64) *p* < .001	PR: 4.35 (2.65–5,81) *p* < .001
White	7 (5.2%)	128 (94.8%)			
Black	13 (65%)	7 (35%)			

*Hb* Hemoglobin Level, *PR* Prevalence ratio, *95% CI* Confidence interval 95%, *AF* Afro-Brazilian, *WH* White, *BL* Black

## Discussion

The finding of lower red blood cell counts in pregnant women with preterm birth corroborates the literature. Anemia during pregnancy affects about 40% of pregnant women, with more than 50% of them due to iron deficiency [11, 12]. Therefore, it deserves a lot of attention, since it can be a risk situation for the mother and the baby. We too observed that women with preterm labor haved high monocytes absolute count, indicating that the peripheral circulatory system maybe has been activated. These increased levels could, perhaps, be used as a marker to predict preterm delivery [13, 14].

The most important clinical characteristics of postpartum women is the HDP, responsible for the largest number of neonatal complications such as increased prematurity and low birth weight [15]. Postpartum women or pregnant with HbAS hemoglobin were asymptomatic most of the time and did not present severe clinical and comorbid conditions [16]. However, in patients with HbSS (sickle cell anemia), maternal-fetal morbidity rates are high and the main clinical complications in these cases are the high incidence of prematurity, numbers of miscarriages and intrauterine fetal death [17].

Current studies involving structural hemoglobinopathies in pregnant women and newborns have shown that most of them go through uninterrupted pregnancies [18, 19]. However, we do not have the ability to predict the clinical direction of the patient during gestation, since the clinical management used in prenatal care is still the determining factor for avoiding serious clinical complications [20].

The prevalence of abnormal hemoglobin found in newborn at IMDL was similar to that reported by Naoum (1997) [21]. For the sickle cell trait, the prevalence of 1.94% (16/825) in our study corresponds to the average national prevalence of sickle cell trait in Brazil, which is 2.1%, though with regional variations reaching values above 5% [22]. According to the National Agency of Sanitary Surveillance, there are approximately 2 million heterozygous (HbAS) individuals [23]. The highest frequencies in Brazil for abnormal hemoglobin are found in the states with the highest concentration of Afro-descendants such as Bahia and Rio de Janeiro, for example [24]. The northeastern region presents the highest frequencies, with the state of Bahia showing the highest prevalence; around 5.55% of the 1422 individuals analyzed [25].

Just abnormal hemoglobins S and D found were in the newborns from Manaus. Interestingly, hemoglobin D is highly prevalent in India, whereas HbS and HbC are prevalent in Africa. Although HbC in homozygosis does not lead to a serious clinical condition such as HbSS, it is clinically important when in dual heterozygosis with HbS, and contributes to an increase in clinical manifestations, similar to what occurs in sickle cell anemia patients [26, 27]. Thus, the finding of heterozygotes for HbD in our population should be considered as an important epidemiological fact. Our results with the predominance of HbS and HbD in Afro-descendants, especially in heterozygous newborns, are explained by the evident racial miscegenation that occurred in our state [28]. This miscegenation caused the HbS to cease to be a characteristic restricted to the black ethnicity population, being frequently found also in admixed population populations, corroborating with our results in which the admixed ethnicity has the second largest number of heterozygous carriers for HbS [29].

The frequency of variant hemoglobins in our study is similar to previous studies carried out in the northern region of Brazil. In the Brazilian states of Rondônia and Acre, the proportion of live births with the sickle trait are 1:250 and 1:3840, respectively, as per the data from the National Neonatal Screening Program [30]. Siqueira et al. (2009) [31] reported a frequency of 2.9% for HbAS and 0.04% for HbSS in the state of Rondônia, while Cardoso et al. (2012) [7] presented a frequency of 1.7% for sickle cell trait (AS) and zero (0%) for HbC in Santarém, Pará state, Brazil. Other studies as Souza et al. (2010) [32] presented an incidence of 1.37% for HbAS, 0.37% for HbAC and 0.007% for HbAD, in the city of Dourados, Mato Grosso do Sul, while Cândido-Bacani et al. (2022) [33] described 2.6% of newborn samples from the Neonatal Screening Center in the state of Mato Grosso do Sul, Brazil, being 75.4% heterozygous for HbAS and 24.6% heterozygous for HbAC. Studies in other Brazilian states, Pinheiro et al. (2006) [34] found a frequency of 3.84% for FAS and 0.93% for FSS from umbilical cord blood samples in the city of Fortaleza; Diniz et al. (2009) [35] reported an FAS profile frequency of 3.23% in newborns in Brasília; Carlos et al. (2018) [36] related FAS frequency of 4.58% from newborns in Minas Gerais; Adorno et al. (2005) [37] described an FAS frequency of 9.8% in newborns from a maternity hospital in Bahia, which is considered to be one of the largest frequencies found in Brazil.

Data from 2013 by IBGE (https://biblioteca.ibge.gov.br/visualizacao/livros/liv63405.pdf) confirms that the northern region, specifically the state of Amazonas, has the highest prevalence of the African-Brazilian population with 77.2% followed by Pará (72.6%), Acre (67.7%) and Amapá (66.9%). Silva et al. (2020) [38] affirmed that in Pará there is a greater concentration of self-identifying black and African-Brazilian, with a prevalence of 4.4% for HbAS individuals and 1% for HbSS. It is known that the process of miscegenation can be analyzed from the point of view of geographic distribution, in which the interior of the northeast and the extreme north of Brazil (states of Amazonas, Pará, and Maranhão) were formed by the process of miscegenation white Europeans and indigenous populations, which can also be observed in the states of Mato Grosso, Mato Grosso do Sul and Goiás [39–41].

As expected, no difference was observed between the presence of FAS or FAD between genders of neonates born at IMDL. Moreover, we found no statistical differences between hematological, biochemical and clinical data regarding gender between the newborns FAA profile and the other heterozygotes. Evidently, studies have shown that there is no correlation between the prevalence of abnormal hemoglobins and the gender of individuals, since the gene responsible for this disease is not linked to sex [42]. In addition, research shows that there is no statistically significant difference in the newborn’s weight and Apgar score, when they have hemoglobin in heterozygosis, such as HbAS, HbAC and HbAD [43]. Low birth weight has been reported in a small number of cases among children of mothers with “S” stroke. However, studies carried out afterwards have not confirmed these findings [44].

Our results showed that neonate carriers of FAS and FAD hemoglobin do not demonstrate a greater tendency for intercurrent neonatal diseases. This fact can be explained by the predominance of fetal hemoglobin, with Hb S being reduced to around 10% in quantity, and therefore unable to cause problems in the immediate neonatal period [45–47]. The incidences found of preterm and low birth weight infants and perinatal mortality were not different between postpartum women who had newborns the FAS profile and those with the FAA profile. There was no difference too in mean birth weight of newborns at IMDL with and without FAS or FAD. Adorno et al. (2005) [37], in their study of a maternity hospital in Bahia, Brazil did not report any difference in the weight of newborns that were carriers of abnormal hemoglobin and newborns that were not carriers. Okonofua et al. (1990) [48] made a comparison between pregnant women from Nigeria with HbAS and FAA and found no difference in the mean weight of newborns between the two groups.

The newborns from IMDL with the FAS and FAD profiles showed expected laboratory profiles that were characterized by normal levels for hematological data. Erythrocyte counts and blood cell morphology are generally normal in patients with this condition. The survival of the red blood cells is also normal, and it is rarely associated with significant clinical or hematological manifestations; the individuals do not present anemia, and hemoglobin varying between 13 and 16 g/dL, therefore it is a benign condition [49–51].

During pregnancy, some studies associate the presence of some comorbidities such as splenic infarction, pyelonephritis and bacteriuria, hyposthenuria and hematuria in pregnant women with sickle cell trait. Yet, pregnancy-specific pneumonia and hypertensive disease are described as common clinical manifestations in pregnant women HbAS [51–53]. In the results of the mothers, it was also possible to identify that the main clinical manifestations were urinary tract infections (UTI) and HDP. Bonamigo et al. (2011) found that the greatest number of intercurrent diseases in the neonatal period were in hematologic systems (90.7%) and respiratory cases (85%). These insufficiencies contributed to the increase in hospitalizations in the NICU, especially in premature infants [54].

Newborn weight can be considered an important clinical indicator as it reflects maternal health conditions. According to the WHO, birth weight of less than 2500 g is defined as low birth weight [55]. Its determinants are prematurity and restriction of uterine growth. Our results corroborate with that of Maia et al. (2010) who identified that 9.13% of preterm newborns have low birth weight [56]. Other study shows to prematurity as one of the most harmful conditions for the development of the newborn baby [57–59]. There is great difficulty in associating clinical findings and laboratory alterations with the probability of a new interruption and the main causes associated with recurrent miscarriage have been extensively discussed in the world literature [60, 61]. However, several studies address that genetic, hormonal and immunological changes are the main factors that predispose to recurrent miscarriage [62–64]. High cholesterol in pregnancy is a major risk for the mother and fetus [65]. It is normal during pregnancy to increase cholesterol and triglyceride levels, mainly attributed to hormonal changes that occur in the pregnant woman’s body. Distinguishing between primary and secondary dyslipidemia is not straightforward, since most dyslipidemias are polygenic and result from a combination of genetic and non-genetic factors [66, 67]. In our study, however, the elevation in the postpartum women of serum lipids was not associated with impairments on the variables clinical, hematological and biochemical parameters of the newborns. Our data showed that pregnant women who underwent prenatal care had the lowest frequency of low birth weight and prematurity, corroborating with Aragão et al. (2004) who verified that one of the risk factors for prematurity was the non-attendance of the mothers to prenatal examinations [68].

The literature shows that, compared to whites, African-Americans have lower average hemoglobin and lower hematocrit levels and mean corpuscular volume (MCV) lower [69, 70]. These data indicate that the problem cannot be solved by simply establishing different levels for different ethnic groups, especially since all groups possess some degree of admixture. Thus, it is basically information that the physician must consider and that becomes one the many factors that we leave to clinical judgment [71, 72]. While it is customary to apply the same hematological reference levels to individuals with diverse ethnicity origins, the prevalence of anemia is more consistent in black and African-Brazilian then white populations, particularly in the individuals with European or African ancestry [73].

The data obtained show a prevalence of hemoglobin or hemoglobinopathies compatible with the estimates for the state of Amazonas. The similarity of the presence of Hb S or Hb D between African-Brazilian and white neonates suggests that screening based on skin color is not indicated. This fact is reinforced by the non-interference of the presence of Hb S or D in the conditions of birth, which corroborates with the recommendation of neonatal screening of hemoglobinopathies as a universal practice.

This research aimed to present the prevalence of abnormal hemoglobin in newborns from the State of Amazonas, and the results obtained reinforce the need to implement educational and prevention programs to raise awareness among the population, in order to reduce the effects caused by these genetic differences and contribute to the improvement of the quality of life of these patients. Through this study, it was possible to estimate, for the first time, the prevalence of structural hemoglobinopathies in newborns of a public maternity unit in Manaus, Amazonas state, Brazil and the possible correlations with hematological, biochemical and clinical data of this population.

Furthermore, the present study emphasizes the need for neonatal screening with the follow-up of positive cases and genetic counseling of affected families. It also suggests inclusion of testing for hereditary anemia as a routine prenatal exam, in order to counsel parents regarding the probability of having a child with abnormal hemoglobins homozygous as HbSS, HbCC or HbDD.

## Conclusion

Based on the objectives proposed in this study, and the methodologies used for the investigation of structural hemoglobinopathies in newborns of a public maternity hospital in the state of Amazonas, we conclude that:

- The frequency of structural hemoglobinopathies was higher in African-Brazilian newborn babies;
- Newborns that are carriers of FAS and FAD showed normal hematological and biochemical parameters and normal birth weight;
- Newborn infants with low birth weight had a higher frequency of intensive care admissions (NUCI);
- Underage mothers had a higher frequency of newborn with low birth weight and premature birth;
- Postpartum women who had children carriers of FAS and FAD had a higher frequency of urinary tract infection and anemia.

## Supplementary Information

**Additional file 1: Supplementary Table 1.** Clinical event in postpartum women by newborns weight attended at IMDL/Manaus-AM (March, 2014 – January, 2015).

## Data Availability

We consider that this original article has no conflict of interest, once that it does not have financial relation with industry, and all subjects involved at the work had the participation allowed by them or by a responsible family member that signed an informed consent. Moreover, this article is original work and that it is not under submission to any other journals. The full data used to support the findings of this study, including, consent terms, electronic files, as well as the lab techniques and materials used may be released upon reasonable request to my Institutional email address: jpmn@ufam.edu.br.

## Abbreviations

IMDL: Instituto da Mulher Dona Lindu
AF: Afro-Brazilian
WH: White
BL: Black
UTI: Urinary tract infections
NICU: Neonatal intensive care unit
HbC: Hemoglobin C
HbS: Hemoglobin S
FAA: Normal Hemoglobin AA
FAD: Heterozygous for D hemoglobin
FAS: Sickle cell trait
Hb: Hemoglobin Level
HDL: High-density lipoprotein
HDP: Hypertensive disorders of pregnancy
HDP: Hypertensive disorders of pregnancy
HPLC: High-performance liquid chromatography
GGT: Gamma glutamyl transpeptidase
LDH: Lactate dehydrogenase
LDH: Lactate dehydrogenase
BI: Indirect bilirubin
DB: Direct bilirubin
TB: Total bilirubin
IBC: Iron binding capacity
MCH: Mean corpuscular hemoglobin
MCHC: Mean corpuscular hemoglobin concentration
MCV: Mean corpuscular volume
MPV: Mean platelet volume
RBC: Red blood cell
RDW: Red blood cell distribution width
χ2: Chi-square
95% CI: Confidence interval 95%
PR: Prevalence ratio
SD: Standard Deviation
WHO: World Health Organization
