# Supplementary material for: Basic biochemical and hematological parameters of structural hemoglobin variants in the postpartum women and their respective newborn from Manaus, Amazonas, Brazil
Source: BMC Pregnancy Childbirth. 2022 Dec 15;22:936. doi: 10.1186/s12884-022-05143-7 (PMC9756781; doi:10.1186/s12884-022-05143-7)
Supplement: Supplementary file 1 — Additional file 1: Supplementary Table 1. Clinical event in postpartum women by newborns weight attended at IMDL/Manaus-AM (March, 2014 – January, 2015). [file 12884_2022_5143_MOESM1_ESM.doc]

Supplementary Table 1. Clinical event in postpartum women by newborns weight attended at IMDL/Manaus-AM (March, 2014 – January, 2015).

| CLINICAL EVENT |  | Weight < 2500kg | Weight ≥ 2500kg | PR (95% CI) | p-value |
| --- | --- | --- | --- | --- | --- |
| HDP | Yes | 10 (25%) | 30 (75%) | 3.61(1.81-6.51) | <.001 |
|  | No | 54 (6.9%) | 726 (93%) |  |  |
| Previous History of Miscarriage | Yes | 31 (23.84%) | 99 (76.16%) | 6.09 (3.65-10.14) | <.001 |
|  | No | 27 (3.91%) | 663 (96.09%) |  |  |

HDP: hypertensive disorders of pregnancy PR: prevalence ratio 95% CI: conﬁdence interval 95%
